# Essential updates 2017/2018: Recent topics in the treatment and research of gastric cancer in Japan

**DOI:** 10.1002/ags3.12284

**Published:** 2019-08-27

**Authors:** Shuhei Komatsu, Eigo Otsuji

**Affiliations:** ^1^ Division of Digestive Surgery Department of Surgery Kyoto Prefectural University of Medicine Kyoto Japan; ^2^ Department of Surgery Kyoto First Red Cross Hospital Kyoto Japan

**Keywords:** biomarker, gastric cancer, Gastric Cancer Treatment Guidelines, Japanese Classification of Gastric Carcinoma, PD‐1 immune checkpoint inhibitor

## Abstract

Recent developments in diagnostic technology, accumulated clinical effort and established evidence have boosted early detection and drastically improved early and long‐term outcomes of gastric cancer. However, gastric cancer continues to be one of the most aggressive and life‐threatening malignancies among all cancers and is a global health problem. Between January 2017 and December 2018, various fascinating reports of managements and research were published, including the new 15th Japanese Classification of Gastric Carcinoma reflecting the 8th American Joint Committee on Cancer/Union for International Cancer Control (AJCC/UICC) tumor, node and metastasis (TNM) classification (October 2017) and the new Gastric Cancer Treatment Guidelines version 5 (January 2018). Moreover, pivotal molecular features of gastric cancer were clarified by the worldwide cancer genome project, and various treatment targets and biomarkers such as circulating DNAs and microRNAs were detected. Novel treatment options using programmed cell death protein 1 immune checkpoint inhibitors have been started. In this review, we summarize the recent topics of classification, guidelines, and clinical and basic research in order to bring new insights to gastric cancer treatment.

## Abstract 2

The greatest change in the 15th edition of JGCA staging systems for gastric cancer was the separate inclusion of N3a and N3b. In the new 15th JGCA classification, T1–T3 disease was upstaged with N3b, T4aN3a was downstaged from IIIC to IIIB and T4bN0 and T4aN2 were downstaged from IIIB to IIIA.

## Graphical abstract

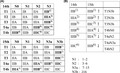

## 1 INTRODUCTION

Gastric cancer is considered to be the fifth most common cancer and the third‐leading cause of death worldwide.^1^ Although recent developments of diagnostic technology, accumulated clinical effort and, thereby, established evidence have boosted early detection and drastically improved the early and long‐term outcomes of gastric cancer, gastric cancer still continues to be a global health problem and causes various clinical treatment problems.^2^ Between January 2017 and December 2018, various fascinating reports were published, including the new 15th Japanese Classification of Gastric Carcinoma reflecting the 8th American Joint Committee on Cancer/Union for International Cancer Control (AJCC/UICC) tumor, node and metastasis (TNM) classification (October 2017)^3^ and the new Gastric Cancer Treatment Guidelines version 5 (January 2018).^4^ In this review, we summarize the topics of these reports related to new classifications, treatment guidelines, clinical research such as chemotherapy, nutrition and pathophysiology and basic research such as molecular features and liquid biopsy in gastric cancer.

## 2 CURRENT STATUS AND TOPICS OF THE 15TH JAPANESE CLASSIFICATION OF GASTRIC CARCINOMA BY THE JAPAN GASTRIC CANCER ASSOCIATION (JGCA)

### 2.1 New 15th JGCA and 8th AJCC/UICC TNM staging related to definition of N3

**Figure 1:**
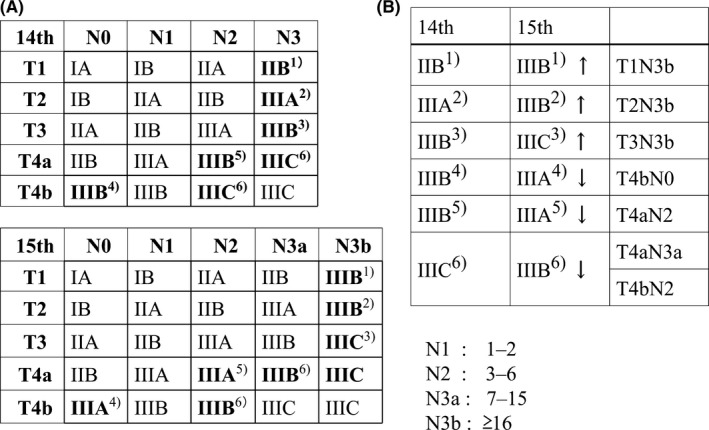
Stage distribution of the 14th Japanese Gastric Cancer Association (JGCA) and the new 15th JGCA classifications. A, The greatest change in the 15th edition of JGCA staging systems for gastric cancer was the separate inclusion of N3a and N3b. B, In the new 15th JGCA classification, T1‐T3 disease was upstaged with N3b, T4aN3a was downstaged from IIIC to IIIB and T4bN0 and T4aN2 were downstaged from IIIB to IIIA

Tumor‐node‐metastasis (TNM) classification systems have been the most important tool for cancer treatment and evaluation of patient outcomes worldwide. Although many studies have confirmed that the 7th edition of the AJCC/UICC TNM classification had a higher prognostic predictive ability than its previous TNM systems,^5^ some limitations remained because N3a (7‐15 metastatic lymph nodes) had the same staging as N3b (≥16 metastatic lymph nodes).^6^ However, the greatest change in the recently released 8th edition of AJCC/UICC TNM and the 15th edition of JGCA staging systems for gastric cancer was the separate inclusion of N3a and N3b.^7^ Therefore, the new 15th JGCA^3^ and the 8th AJCC/UICC TNM classifications differed in that T1‐T3 disease was upstaged with N3b, T4aN3a was downstaged from IIIC to IIIB, and T4bN0 and T4aN2 were downstaged from IIIB to IIIA (Figure 1).^3^ The 8th edition was validated as showing better prognostic ability than the 7th edition and had a good concordance index (C‐index, 0.719), which was comparable to that of the 8th edition developed from the International Gastric Cancer Association (IGCA) data (0.775).^8^ Particularly, these tendencies were precisely proven in patients with >15 retrieved lymph nodes (RLN), because a stage migration may occur if a low number of lymph nodes are retrieved, which may underestimate the malignant potential of gastric cancer.^8^, ^9^

Number of RLN is influenced by various factors: extent of lymphadenectomy, enthusiasm to examine more lymph nodes pathologically and the surgical situation, such as fat volume and the innate number of lymph nodes in each patient. Although at least 16 RLN are recommended for staging in the JGCA and AJCC/UICC TNM classifications, a significant prognostic difference has been reported even between RLN <16 and RLN ≥16 in patients with pStage II‐III gastric cancer.^10^ Previous studies have identified that RLN ≥25 could eliminate the prognostic effect^10^ and presented better prognostic staging.^11^ Indeed, the German S3 guidelines also recommend ≥25 RLN as a criterion for a D2 gastrectomy. Therefore, RLN ≥25 may be needed for precise nodal staging in the new classification.

Nevertheless, a low number of RLN may still be influenced by various situations and thereby be imperative. Positive lymph node ratio (PLNR), which is obtained by dividing the metastatic lymph node count by the RLN count, has been proposed as an alternative and putative superior staging method to avoid stage migration^12^, ^13^, ^14^ and could evaluate the quality of TNM staging.^15^ RLN ≥8 might be sufficient as an appropriate use of the PLNR system.^13^

### 2.2 New definition of the esophagogastric junction (EGJ) and distribution of esophageal cancer and gastric cancer in EGJ adenocarcinoma

Definition of EGJ and staging of the carcinoma of EGJ have not been standardized internationally. A unified definition was decided in both the 15th JGCA and the 11th Japan Esophageal Society (JES) classifications, compared to the previously published AJCC/UICC TNM classification. Specifically, the 15th JGCA^16^ and the 11th JES classifications^17^ defined the EGJ as the border between esophageal and gastric muscles to be defined from the distal end of longitudinal palisading vessels in the lower esophagus to the proximal end of the longitudinal gastric folds. The priority of the EGJ definition was the distal end of longitudinal palisading vessels by the endoscopic findings. Regarding the definition of EGJ cancer, the Nishi classification defined EGJ carcinoma as a tumor located in the area from 2 cm above to 2 cm below the EGJ irrespective of histological type. Whereas, the Siewert classification covering 5 cm above and below the EGJ is widely used to classify EGJ adenocarcinoma, although a strict definition of EGJ itself has not been described.

In the 7th AJCC/UICC TNM classification, adenocarcinoma arising within 5 cm below the EGJ, which is defined as Siewert I to III, was staged as an esophageal cancer. However, in the new 8th AJCC/UICC TNM classification, EGJ adenocarcinoma such as Siewert I and II was defined as an esophageal cancer, for which the tumor is located from 5 cm above to 2 cm below the EGJ, whereas EGJ adenocarcinoma such as Siewert III was defined as a gastric cancer, for which the tumor is located from 2 cm below to 5 cm below the EGJ.^7^

### 2.3 New definition of subclassified station no. 6 lymph nodes and new allocation of station no. 13 regional lymph nodes as a regional lymph node in duodenal invasion of gastric cancer

Infrapyloric station no. 6 lymph nodes were anatomically subclassified into three regions as follows: no. 6a, lymph nodes along the right gastroepiploic artery; no. 6i, lymph nodes along the infrapyloric artery; and no. 6v, lymph nodes on the anterior surface of the pancreatic head along the right gastroepiploic vein and the infrapyloric vein.^16^, ^18^ This subclassification is anatomically useful to carry out antrum‐preserving gastrectomy. Based on this definition, a prospective observation study identified that the metastasis rate to no. 6i nodes was 2.1% in early lower‐third tumors and 19.5% in advanced tumors.^19^ Moreover, in patients with positive no. 6i nodes, the distance from the distal tumor border to the pyloric ring was proven to be within 44 mm. In contrast, no early middle‐third gastric cancers had no. 6i metastasis.^19^

According to the TNM classification, in the rare occurrence in which a tumor involves more than one organ or structure, the regional nodes have been reported to include those of all involved structures, even if the nodes of the primary site are not involved. Specifically, in the esophageal invasion of gastric cancer, station no. 19, 20, 110 and 111 lymph nodes were considered to be regional lymph nodes.^16^ Also, in the duodenal invasion of gastric cancer, a station no. 13 lymph node, which was defined as a lymph node located in the posterior surface of the pancreatic head, was considered to be a regional lymph node but not a distant lymph node.^16^

### 2.4 Miscellaneous

One of the biggest changes in the new 15th JGCA and 8th AJCC/UICC TNM staging is a separation of clinical TNM and pathological TNM stages. Clinical stage is simplified (Table 1). Namely, clinical tumor depth staging was divided into three degrees such as: (i) T1, T2; (ii) T3, T4a; and (iii) T4b. Also, nodal staging was divided into two degrees such as: (i) N0; and (ii) N1, N2, N3. Regarding other changes, the definition of peritoneal metastasis is important for current treatment strategies such as diagnostic laparoscopy and conversion surgery for stage IV gastric cancer following chemotherapy. The new revision of macroscopic peritoneal metastasis was as follows: PX, peritoneal metastasis is unknown; P0, no peritoneal metastasis; and P1, peritoneal metastasis. P1 was subclassified into P1a, P1b, P1c and P1x according to the sites of peritoneal dissemination. Also, the new revision of lymphatic invasion was as follows: Ly0, lymphatic invasion is negative; and Ly1, lymphatic invasion is positive. Ly1 was subclassified into Ly1a, Ly1b, Ly1c according to the extent of lymphatic invasion. Venous invasion was classified the same way. Regarding the residual tumor (R) concept, R concept is to be used only in surgical resection. In the pathological evaluation after endoscopic resection, R concept is not used for the status of vertical and horizontal margins.^16^ Namely, the concept of curability after endoscopic resection was defined in the new Japanese Gastric Cancer Treatment Guidelines version 5 (2018).

**Table 1:** Clinical staging in the new 15th JGCA and 8th AJCC/UICC TNM classifications

	N0	N1, N2, N3
T1, T2	I	IIA
T3, T4a	IIB	III
T4b	IVA	
M1 with any T/N	IVB	

Abbreviations: AJCC/UICC, American Joint Committee on Cancer/Union for Cancer Control; JGCA, Japanese Gastric Cancer Association; TNM, tumor, node and metastasis.

## 3 CURRENT TOPICS OF THE JAPANESE GASTRIC CANCER TREATMENT GUIDELINES 2018 (VERSION 5)

### 3.1 Recent overview of topics from the latest version

The latest version of the Japanese Gastric Cancer Treatment Guidelines (January 2018) contains a novel algorithm for gastric cancer.^4^ Briefly, the committee added novel treatment indications for stage IV gastric cancer, EGJ carcinoma, standard D2 gastrectomy, neoadjuvant and adjuvant chemotherapy and others. Also, the committee established a Q & A section to provide tentative best answers to important clinical questions. Major categories were as follows: (i) clinical questions of surgical resection (CQ1‐CQ10); (ii) clinical questions of endoscopic treatments (CQ11‐CQ12); (iii) clinical questions for non‐resectable or recurrent gastric cancer (CQ13‐CQ22); and (iv) clinical questions of perioperative chemotherapy (CQ23‐CQ26).

### 3.2 Clinical indications of stage IV gastric cancer

Regarding the novel algorithm of version 5 (2018), new treatment guidelines of stage IV gastric cancer were depicted in greater detail than in the previous version. In advanced gastric cancer with a single stage IV non‐curable factor except for cytology positive factor, a recent phase III study (REGATTA study) showed that cytoreductive gastrectomy followed by chemotherapy did not show any survival benefit compared with chemotherapy alone. Therefore, cytoreductive gastrectomy followed by chemotherapy cannot be justified for the treatment of patients with stage IV factors (CQ1).^20^ However, some clinical concerns may remain unanswered for elderly or high‐risk patients who cannot tolerate standard chemotherapy.

Although the REGATTA study indicated demerit for the strategy of palliative gastrectomy followed by chemotherapy, it remains unclear whether conversion surgery with curative intent for stage IV gastric cancer would be justified after complete response for stage IV factors following intensive chemotherapy.^21^ Yoshida et al^22^ recently suggested a comprehensive classification of gastrectomy for stage IV cancer that takes conversion surgery into consideration. Of all enrolled patients, resected patients had a better prognosis than unresected patients (mean survival time (MST), 30.5 months vs 11.5 months). Thus, treatment guidelines also suggested the putative indication (CQ5, CQ8, CQ20) of conversion gastrectomy with curative intent for patients with a single stage IV factor based on previous studies.^4^, ^21^

Regarding para‐aortic lymph node dissection for the no. 16 a2/b1 region in apparently swollen nodes (CQ5), the strategy was explored in the JCOG 0405, 1002 phase II trials,^23^, ^24^ such as absence of peritoneal deposits and negative peritoneal washing cytology through staging laparoscopy, absence of metastasis to other organs, and absence of cancer spread to the a1 or b2 regions and mediastinal/cervical lymph nodes. It is likely that only patients with a moderate number of swollen nodes in the no. 16 a2/b1 region might be considered for conversion surgery following chemotherapy. Concerning hepatic metastasis (CQ8), patients with ≤3 nodules, which are invariably diagnosed using enhanced magnetic resonance imaging (MRI), and without other stage IV factors might be candidates for hepatectomy following chemotherapy because these patients have an outstanding outcome of more than 30% in 5‐year survival.^25^ Nevertheless, a large number of prospective studies would be needed with control arm patients treated only by chemotherapy. Regarding patients with only CY1 factor (CQ20) or minute peritoneal metastasis, which is recognized as a clinical status of micro‐metastasis, and only a low tumor burden at the peritoneal cavity, perioperative S‐1 based chemotherapy for CY1^26^ or HIPEC (hyperthermic intraperitoneal infusion chemotherapy) for CY1^27^ or i.p. and i.v. paclitaxel plus S‐1^28^ in addition to curative gastrectomy could be considered. Intraperitoneal and i.v. paclitaxel plus S‐1 appears to be effective not only for CY1 or low‐tumor‐burden peritoneal metastasis but also for highly advanced peritoneal metastasis cases with malignant ascites. Also, recent studies suggest that i.p. taxane may be useful to control highly advanced peritoneal metastases.^29^

### 3.3 New guidelines for the extent of lymphadenectomy and type of gastrectomy in EGJ carcinoma

Gastric cancer located in the cardia or the EGJ has drastically increased in Asia and South America as well as in the USA and Europe.^30^ There is considerable controversy as to whether EGJ adenocarcinoma in the Nishi classification, which is similar to Siewert type II carcinoma, is actually esophageal cancer or gastric cancer. From the viewpoint of lymphatic spreading, total gastrectomy is apparently a more common procedure than proximal gastrectomy in this population. Nevertheless, in EGJ adenocarcinoma of less than 40 mm, a recent nationwide study identified that the therapeutic value of lymphadenectomy was high at station nos 3, 1, 2 and 7, and the incidences of metastasis at station nos 4sa, 4sb, 4d, 5 and 6 were <1% even in patients with high dissection rates.^31^ Previous studies also showed that the incidences of metastasis at station nos 4d, 5 and 6 were low, and that the benefits of prophylactic nodal dissection of these regions were questionable^32^, ^34^, ^35^, ^36^, ^37^, ^38^ (Table 2). The new guidelines indicated in CQ6 that complete nodal clearance along the distal portion of the stomach offers only a marginal survival benefit, and total gastrectomy is not essential for local control in EGJ adenocarcinoma of <40 mm.

**Table 2:** Therapeutic value of lymphadenectomy in esophagogastric junction adenocarcinoma

Station no.	First author/Year Yamashita et al 2011,^32^ (n = 225)	Mine et al 2013,^34^ (n = 125)	Fujitani et al 2013,^35^ (n = 86)	Yabusaki et al 2014,^36^ (n = 72)	Goto et al 2015,^37^ (n = 92)	Yoshikawa et al 2016,^38^ (n = 381)	Yamashita et al 2015,^33^ (n = 2846)
1	13. 8	18.7	16.3	11.1	14.3	16.2	7.5
2	7	15.3	5.8	8.3	2.1	13.6	4.3
3	13.7	20.7	11.6	11.1	16.8	19.8	9
4sa	1	0	3.5	0	0	1	0.4
4sb	0		1.2	0	1.1	0.3	0
4d	NA		0	1.5	0	1.1	0
5	0	0	0	0	0	0	0
6	0	NA	1.2	1.6	0	0.4	0
7	3.8	14.8	5.9	5.6	8.8	11.7	3.7
8a	2.2	0	2.4	1.5	1.8	0.7	0.8
9	1.5	1.4	1.3	1.4	3.9	1.7	1.4
10	0.7	1.9	1.5	2.3	0	1.8	0.8
11p	2.6	3.8	8.2	1.6	2.5	4.7	1.7
11d	2.2			0	0	1.7	
12a	NA	0	NA	0	0	0	0
19	NA	NA	NA	NA	0	0	0.6
20	NA	NA	NA	NA	4.8	0	0.6
110	1.8	6.3	NA	2.9	NA	6.5	2.9
111	0		NA	0	NA		0.4
112	0		NA	0	NA		1.5
16a2	1.4	3.2	NA	4.8	NA	2.4	0

Abbreviation: NA, not applicable.

### 3.4 New guidelines for standard D2 gastrectomy for advanced proximal gastric cancer

In Japan, total gastrectomy with splenectomy is carried out as a standard D2 procedure for complete removal of the splenic hilar lymph nodes, which are defined as a station no. 10 lymph node. However, a recent Japanese multicenter phase III trial (JCOG0110) compared splenectomy with spleen‐preserving surgery and confirmed the survival non‐inferiority of spleen‐preserving surgery against splenectomy for advanced proximal gastric cancers not invading the greater curvature.^7^ Therefore, the new guidelines suggested that prophylactic splenectomy should be avoided in patients undergoing total gastrectomy for advanced proximal gastric cancer that does not invade the greater curvature, because it increases operative morbidity without improving survival (CQ4). Regarding gastric cancer invading the upper third of the greater curvature, the significance of prophylactic splenectomy remains unclear because there is no clinical evidence from prospective studies. However, a recent retrospective study using a large number patients showed high rates of metastasis to the splenic hilar lymph node and a high therapeutic index.^39^ Therefore, currently, the splenic hilar nodes might be included as a standard component of D2 lymphadenectomy for such tumors.

Regarding bursectomy for resectable cT3‐T4a gastric cancer, it has been carried out as a standard component of D2 gastrectomy during curative distal or total gastrectomy. However, it remains controversial as to whether bursectomy can prevent peritoneal metastasis.^40^, ^41^ A recent prospective JCOG1001 trial showed that bursectomy did not provide a survival advantage over non‐bursectomy.^42^ Therefore, the new guidelines suggested that bursectomy should be avoided as a standard component of D2 gastrectomy. However, D2 dissection with omentectomy is still warranted as standard surgery for resectable cT3‐T4a gastric cancer due to a lack of evidence against it.

### 3.5 New categories for the indications and curability of endoscopic treatment

In the new treatment guidelines, two major revisions were made in the treatments using endoscopy. Indication for endoscopic resection was divided into three categories, which are absolute indication, expanded indication, and relative indication. Also, endoscopic curability was categorized into three types, which are eCuraA, eCuraB, and eCuraC. eCuraC was subcategorized into eCuraC1 and eCuraC2. eCuraA is en bloc resection status of UL0, differentiated type, pT1a, HM0, VM0, Ly0 and V0, or UL1, differentiated type, <3 cm, pT1a, HM0, VM0, Ly0 and V0. eCuraB is en bloc resection status of UL0, undifferentiated type, <2 cm, pT1a, HM0, VM0, Ly0 and V0, or differentiated type, <3 cm, pT1b (SM1), HM0, VM0, Ly0 and V0. eCuraC1 is resection status where en bloc resection or HM0 is not fulfilled by the definition of eCuraA or eCuraB. eCuraC2 is resection status other than eCuraA, eCuraB or eCuraC1.^43^

These definitions were established by expert endoscopists in multiple high‐volume centers using approximately 2000 patients. Therefore, a real‐world validation analysis using the National Clinical Database (NCD), which includes resection data by non‐expert endoscopists, is also needed to investigate whether the eCura system is indeed useful in practical clinical settings. Recently, patients who required radical surgery after endoscopic submucosal dissection for early gastric cancer were investigated.^44^ The eCura system consists of five clinicopathological factors, which are scored as follows: one point each for tumor size >30 mm, positive vertical margin, venous invasion and SM2 (depth of tumor invasion into the submucosa is ≥500 μm from the muscularis mucosa), and three points for lymphatic invasion. Although the rate of lymph node metastasis is already presented in the new guidelines, caution is needed when applying this system because these data have a selection bias using a small number of patients and include only 14.8% of undifferentiated‐type early gastric cancer.^44^ Also, the status of the positive vertical margin may indicate the presence of advanced cancers. Nevertheless, these data might be useful for elderly or high‐risk patients with comorbidities to avoid additional surgery with lymphadenectomy.

### 3.6 New guidelines for chemotherapy for advanced or recurrent gastric cancer patients

Recent topics concerning neoadjuvant chemotherapy for scirrhous‐type gastric cancer such as type 4 or large type 3 gastric cancer (JCOG0501)^45^ and adjuvant chemotherapy for stage III gastric cancer (JACCRO GC‐07)^46^ were previously nicely reviewed.^47^ In the present study, we summarized the new guidelines for chemotherapy for advanced or recurrent gastric cancer patients, and chemotherapeutic regimens were classified into two categories as follows: (i) recommended regimens; and (ii) conditionally recommended regimens. Recommended regimens as a first‐line therapy for HER2‐negative patients included S‐1 plus cisplatin, capecitabine plus cisplatin, S‐1 plus oxaliplatin, capecitabine plus cisplatin, capecitabine plus oxaliplatin and fluorouracil (5‐FU) plus leucovorin (LV) plus oxaliplatin. In HER2‐positive cases, capecitabine plus cisplatin plus trastuzumab and S‐1 plus cisplatin plus trastuzumab were recommended. As a second‐line treatment, weekly paclitaxel plus ramucirumab was recommended. Nivolumab and irinotecan were recommended as a third‐line treatment.

Of note, nivolumab, programmed cell death protein 1 (PD‐1) immune checkpoint inhibitor has been approved in Japan on the basis of a randomized trial, ATTRACTION‐2, showing a significant survival advantage for patients who received nivolumab compared with placebo in the third or later lines of therapy.^48^, ^49^ ATTRACTION‐2 phase III trial showed that the median overall survival (OS) was significantly better for nivolumab (5.26 months for nivolumab vs 4.14 months for placebo) (HR, 0.63; *P *<* *.0001). One‐year OS rate was 26.2% in the nivolumab arm versus 10.9% in the placebo arm. Gastric cancer harbors numerous somatic mutations related to a large number of neoantigens that can activate T cells.^50^ Pembrolizumab is starting to be used for patients with microsatellite instability (MSI‐H). Although these PD‐1 blockades were proven to be effective for gastric cancer, the relationship between programmed cell death ligand 1 (PD‐L1) expression and tumor response is still controversial with respect to nivolumab and pembrolizumab. Namely, nivolumab is effective regardless of PD‐L1 expression on tumor cells and has been used without any restriction by biomarkers. However, regarding pembrolizumab, which shows encouraging antitumor activity, the response is highly correlated with PD‐L1 expression.^51^, ^52^

Regarding immunorelated adverse events (irAE) and predictive value of response, absolute lymphocyte count (ALC) may be one of the optimal indicators. A multivariate analysis in a previous study suggested that patients with an ALC >2000 at baseline had an increased factor of irAE (odds ratio [OR] 1.99) but had a high treatment response.^53^ A neutrophil‐to‐lymphocyte ratio (NLR) of ≥4 has been reported to have a poorer prognosis.^54^ These indicators might be useful for decision‐making regarding the use of PD‐1 immune checkpoint inhibitors; however, further studies are needed to select useful biomarkers.

## 4 RECENT PIVOTAL TOPICS FOR CLINICAL AND BASIC RESEARCH IN GASTRIC CANCER

### 4.1 Recent topics of preoperative nutrition and absorptive disorders following gastrectomy

Regarding preoperative nutrition, prealbumin concentration, which is a rapid turnover protein and a real‐time and more sensitive nutritional indicator than albumin, was proven to be independently correlated with overall morbidity (OR, >22 mg/dL vs <15 mg/L: 1.0 vs 4.5).^55^ Prealbumin could be a pivotal indicator to improve preoperative nutrition and avoid complications.

A major postoperative concern is weight loss, which is associated with marked deterioration in quality of life (QOL), reduced tolerance to chemotherapy^56^ and worsening of the final prognosis.^57^ A recent multicenter randomized control trial (RCT) identified that a regular diet plus an oral elemental nutritional supplement for 6‐8 weeks using 300 kcal/day of Elental (Ajinomoto Pharmaceuticals, Tokyo, Japan), which contains essential amino acids with a low fat content, could significantly attenuate body weight loss following gastrectomy, especially following total gastrectomy.^58^ However, another recent RCT could not show the significance of using immunonutrition based on an eicosapentaenoic acid‐enriched oral nutritional supplement for 3 weeks perioperatively using 600 kcal/day.^59^ The reason for the negative result is that an oral nutritional supplement may give rise to a decrease in oral intake. In contrast, recent studies have suggested that home enteral nutrition for 6 weeks using jejunostomy feeding did not affect oral intake and improved postoperative nutrition after esophagectomy and total gastrectomy in esophagogastric cancer.^60^ Forced nutrition using jejunostomy home feeding may be one of the strategies in high‐risk weight loss patients after surgery.

Regarding postoperative pathophysiology, the influence of non‐physiological food passage after Billroth‐II and Roux‐en‐Y reconstruction is recognized as a potential cause of metabolic and absorption disorders such as iron, calcium and fatty acid deficiencies. Lee et al^61^ demonstrated that the incidence of iron deficiency varies according to the extent of gastrectomy and the reconstruction method selected; iron deficiency was observed more frequently in patients with Billroth‐II reconstruction than in those with Billroth‐I reconstruction after distal gastrectomy. Compared to Roux‐en‐Y reconstruction, Billroth‐I reconstruction might be preferable for the purpose of preventing a decrease in hemoglobin^62^ and bone mineral density^63^ in gastric patients, particularly in older patients.

### 4.2 Recent topics of molecular features and targeted treatments in gastric cancer

The Cancer Genome Project, which was started in 2005 in the USA, contributed to developing The Cancer Genome Atlas (TCGA) and promoting so‐called “precision medicine” for cancers worldwide. TCGA has reported the results of multiplatform sequencing in primary gastric cancers. Specifically, gastric cancer has been divided into four subtypes with MSI, chromosomal instability (CIN), genome stability (GS) and Epstein‐Barr virus (EBV) association.^64^ The MSI subtype represents approximately 22% and is more frequent in distal gastric cancer than in proximal gastric cancer. In contrast, the CIN subtype is more frequent in proximal gastric cancer. Compared with other gastrointestinal cancers, the CIN subtype in gastric cancer tends to have focal region alterations. The GS and EBV subtypes have frequencies of 20% and 9%, respectively. The GS subtype is enriched in a diffuse‐type histology and is molecularly characterized by fewer mutations and less overexpression of epithelial mesenchymal transition (EMT)‐related genes.^64^ In contrast, the CIN subtype is enriched in intestinal histology and is molecularly characterized by *TP53* mutation and RTK‐RAS activation/amplifications. The MSI subtype harbors numerous somatic mutations related to a large number of neoantigens that can activate T cells.^50^ Thus, gastric cancer with MSI responds well to immune checkpoint blockades.^65^

### 4.3 Molecular biomarkers, targets and recent clinical topics regarding MSI in gastric cancer

Understanding the molecular mechanisms of carcinogenesis and identifying the molecular targets for diagnosis and treatment may contribute to the improvement of survival of patients with gastric cancer. Thus, some molecular targets with frequent targets have been identified,^66^ such as gene amplifications of *MET* and *ERBB2*; hypermethylation of *p16*;^67^, ^68^ mutations of *TP53*,* APC* and *E‐cadherin*;^69^, ^70^, ^71^ oncogenic activation of β*‐catenin* and *K‐ras*;^72^ and inactivation of the mismatch repair gene *hMLH1*, which is associated with MSI.^73^ However, in clinical settings, only a few genes have been used as diagnostic biomarkers and/or molecular therapeutic targets.^74^, ^75^ The development of molecular biomarkers and therapy is urgently required.

A recent study identified that dramatic responses to PD‐1 inhibitors such as pembrolizumab were observed in patients with MSI‐high and EBV‐positive tumors, presenting 85.7% of overall response rate (ORR) in MSI‐high and 100% of ORR in EBV‐positive patients, although the frequency of MSI‐high and EBV‐related gastric cancer in a metastatic setting is low, Also, decreased circulating tumor DNA (ctDNA) was associated with improved outcomes.^65^ MSI‐high and EBV could be pivotal biomarkers as a companion diagnosis.

### 4.4 Nucleic acids as liquid biopsy: Circulating DNA as a clinical biomarker and companion diagnosis in gastric cancer

The concept of liquid biopsy has become widely accepted in a clinical setting. Liquid biopsy is a less invasive approach for obtaining genetic and epigenetic aberrations that are closely associated with cancer initiation and progression. Moreover, liquid approaches allow for repeated sampling, and this makes it possible to evaluate the longitudinal evolution of a tumor and its heterogeneous characteristics, which single sampling may fail to capture.^76^ Recently, a liquid biopsy using nucleic acids such as cell‐free DNAs and microRNAs in blood could be realized in clinical settings. We clarified the utility of the digital polymerase chain reaction (PCR)‐based HER2 copy number assay as a liquid biopsy to detect ERBB2 amplification in blood cell‐free DNA for tumors having heterogeneities.^77^ We identified the potential utility of circulating cell‐free DNA to detect EBV‐DNA. Identification of the EBV subtype using liquid biopsy could be useful for real‐time monitoring of tumor progression and treatment response.^78^ Also, a recent study identified a blood test, Cancer SEEK, that can detect eight common cancer types including gastric cancer through assessment of the levels of circulating proteins and mutations in cell‐free DNA.^79^

### 4.5 Nucleic acids as liquid biopsy: Circulating microRNAs as a clinical biomarker and future perspectives in gastric cancer

MicroRNAs (miRNA), which are small non‐coding RNAs, regulate the translation of specific protein‐coding genes. Altered expressions of miRNAs contribute to the development of gastric cancers. In gastric cancer, various cancer‐related miRNAs and their target genes were detected (Table 3). Also, various circulating miRNAs have already been proven to have the potential to enable diagnosis of gastric cancer at an early stage, predict prognosis and recurrence, evaluate patient status and therapeutic efficacy and provide optimal, individualized treatment strategies in gastric cancer.^80^ As a next‐generation liquid biopsy biomarker reflecting tumor dynamics, particularly, the upregulated oncogenic miRNAs in blood such as miR‐20a, miR‐21, miR‐25, miR‐106a, miR‐106b, miR‐199a‐3p, miR‐221, miR‐223 and miR‐421, might be useful candidates as blood‐based biomarkers for gastric cancer. Regarding the downregulated tumor suppressor miRNAs in blood, let‐7a, miR‐101, miR‐181b, miR‐203, miR‐204, miR‐218, miR‐375, miR‐451 and miR‐486 might be useful candidates as blood‐based biomarkers and oligonucleotide therapeutics for gastric cancer. Liquid biopsy using circulating tumor cells and cell‐free nucleic acids such as cell‐free DNAs and miRNAs in gastric cancer patients could provide valuable new insights into prognosis and treatments in the near future.

**Table 3:** Cancer‐related microRNAs and their target genes in gastric cancer

Overexpression in gastric cancer tissue			Underexpression in gastric cancer tissue		
microRNA	Value^a^	Target gene	microRNA	Value^a^	Target gene
miR‐20a^b^	D, M, P	FBXO31	let‐7 family^b^	D	RAB40C, MYH9, HMGA2, BARX1
miR‐21^b^	D, M, P	PDCD4, RECK	miR‐9		GRB2, NF‐κB
miR‐23a		IL6R	miR‐29		CDC42
miR‐25^b^	D	FBXW7, TOB1	miR‐34		BCL2, NOTCH, HMGA2
miR‐27		Prohibin	miR‐43c		VEZT
miR‐103		DICER1	miR‐101^b^	D, M, P	EZH2, COX2, MCL‐1, FOS
miR‐106a^b^	D	RB1	miR‐125a		ERBB2
miR‐106b‐25 cluster^b^	D, P	p21, p57, Bim	miR‐126		CRK, SOX2
miR‐130b		RUNX3	miR‐129		Cdk6, SOX4
miR‐146a		IRAK1, TRAF6	miR‐137		CDC42
miR‐150		EGR2	miR‐139		CXCR4
miR‐199a^b^	D, P	MAP3K11	miR‐141		FGFR2
miR‐215		ALCAM	miR‐148a		CCKBR, P27, PIN
miR‐221‐222 cluster^b^	D, P	PTEN, P27, p57	miR‐148b		CCKBR
miR‐223^b^	D	FBXW7, STMN1	miR‐152		CCKBr
miR‐372		LATS2	miR‐155		PKIα, MYD88, SMAD2, FADD, IKK‐ε
miR‐421^b^	D	CBX7, RBMXL1	miR‐181b^b^	D	BCL2
miR‐650		ING4	miR‐181c		NOTCH4, KRAS
			miR‐200		ZEB1, ZEB2
			miR‐203^b^	D, M, P	ERK1/2
			miR‐204^b^	M, P	EZRIN
			miR‐212		MECP2
			miR‐218^b^	D, P	NF‐κB, COX2, ROBO1, ECOP
			miR‐331‐3p		E2F1
			miR‐335		BCL‐W, SP1
			miR‐375^b^	D	JAK2, PDK1, YWHAZ
			miR‐429		C‐MYC
			miR‐433		GRB2
			miR‐449		GMNN, MET, CCNE2, SIRT1
			miR‐451^b^	D	MIF
			miR‐486^b^	D	OLMF4
			miR‐497		BCL2
			miR‐512‐5p		Mcl‐1, JUN
			miR‐622		ING1

a Value of liquid biopsy; D, diagnostic value; M, malignant potential value; P, prognostic predicting value.

b Reported blood‐based microRNAs reflecting tumor dynamics in gastric cancer.

## 5 CONCLUSIONS

Thanks to essential updates between January 2017 and December 2018, recent pivotal topics in the classifications, guidelines, treatment management, molecular features and basic research of gastric cancer have been clarified. These updates could provide essential and new insights into gastric cancer treatments in practical clinical settings. However, in the present review, we could summarize only some of the major topics and had to exclude topics on less invasive surgeries such as laparoscopic^81^, ^82^ and robotic gastrectomy and clinical study regarding the surgical approach based on the sentinel node concept for early gastric cancer.^83^ However, additional updates on major topics including these topics could be summarized in the near future.

## DISCLOSURE

Conflicts of Interest: Authors declare no conflicts of interest for this article.
